# Multiple treatments with human embryonic stem cell-derived mesenchymal progenitor cells preserved the fertility and ovarian function of perimenopausal mice undergoing natural aging

**DOI:** 10.1186/s13287-024-03684-6

**Published:** 2024-03-03

**Authors:** Eun-Young Shin, Suji Jeong, Jeoung Eun Lee, Dong Seok Jeong, Dong Keun Han, Seok-Ho Hong, Dong Ryul Lee

**Affiliations:** 1 Department of Biomedical Science, CHA University, 335 Pangyo-ro, Bundang-gu, Seongnam- si, 13488 Gyeonggi-do Republic of Korea; 2 Department of Internal Medicine, School of Medicine, Kangwon National University, Chuncheon, 24431 Gangwon-do Republic of Korea; 3 CHA Advanced Research Institute, Bundang CHA Medical Center, 335 Pangyo-ro, Bundang- gu, Seongnam-si, 13488 Gyeonggi-do Republic of Korea

**Keywords:** Human embryonic stem cell-derived mesenchymal progenitor cells (hESC-MPCs), Ovarian function, Reproductive senescence, Fertility preservation of natural aging female, Myeloid-derived suppressor cells (MDSCs)

## Abstract

**Objectives:**

Currently, no approved stem cell-based therapies for preserving ovarian function during aging. To solve this problem, we developed a long-term treatment for human embryonic stem cell-derived mesenchymal progenitor cells (hESC-MPCs). We investigated whether the cells retained their ability to resist ovarian aging, which leads to delayed reproductive senescence.

**Materials and methods:**

In a middle-aged female model undergoing natural aging, we analyzed whether hESC-MPCs benefit the long-term maintenance of reproductive fecundity and ovarian reservoirs and how their transplantation regulates ovarian function.

**Results:**

The number of primordial follicles and mice with regular estrous cycles were increased in perimenopausal mice who underwent multiple introductions of hESC-MPCs compared to age-matched controls. The estradiol levels in the hESC-MPCs group were restored to those in the young and adult groups. Embryonic development and live birth rates were higher in the hESC-MPC group than in the control group, suggesting that hESC-MPCs delayed ovarian senescence. In addition to their direct effects on the ovary, multiple-treatments with hESC-MPCs reduced ovarian fibrosis by downregulating inflammation and fibrosis-related genes via the suppression of myeloid-derived suppressor cells (MDSCs) produced in the bone marrow.

**Conclusions:**

Multiple introductions of hESC-MPCs could be a useful approach to prevent female reproductive senescence and that these cells are promising sources for cell therapy to postpone the ovarian aging and retain fecundity in perimenopausal women.

**Supplementary Information:**

The online version contains supplementary material available at 10.1186/s13287-024-03684-6.

## Introduction

The ovary has two main roles: the female reproductive organ and a hormone secretion system. The ovaries function cyclically; in humans, their function declines with age from the mid-30s, and their menopause-disrupting endocrine function starts in the mid-50s [1]. Ovarian function declines early relative to other organs in the body and is implicated in predicting ovarian menopause [2]. The mechanism underlying the age-related reduction in ovarian function is a major question in reproductive medicine and has been investigated for several decades. In addition to the loss of ovarian reproductive function, menopause is an unavoidable part of aging and leads to the deterioration of general health by increasing the risk of cardiovascular disease, osteoporosis, vasomotor symptoms, depression, and cognitive impairment [3]. Reproductive aging and menopause are increasingly significant health issues because more women plan to delay childbearing, and their life expectancy has increased [4]. However, although regenerative medicine using various stem cells has been applied to maintain the ovarian reservoir and to extend reproductive potential, effective clinically applicable protocols have not been identified.

Mesenchymal progenitor cells/mesenchymal stem cells (MPCs/MSCs) derived from adult and fetal tissues have been proposed as new therapeutic sources for treating various diseases, including reproductive senescence [5, 6]. MPCs secrete multiple cytokines and growth factors that regulate immune reactions, apoptosis, survival, and cell proliferation [7]. However, the application of these tissue-specific MPCs still has some drawbacks; for example, the harvesting of MPCs requires invasive surgical procedures that may cause severe side effects, and the manufacturing protocol for MPCs is difficult to standardize because of donor heterogeneity. Furthermore, MPCs have a limited cell growth capacity in culture; therefore, it is difficult to obtain sufficient quantities for clinical use [8–10].

Recently, human embryonic stem cell-derived MPCs (hESC-MPCs) have been suggested as an alternative source of MPCs owing to their high proliferative capacity and ease of standardization. Despite the many advantages of hESC-MPCs, there is a risk of developing tumors if undifferentiated hESCs are not completely removed. Many studies are being conducted to overcome this, and our group has overcome this problem by the clonal expansion of MPCs from a single hESC-MPC using their high proliferative power. Single cell-derived clonally expanded hESC-MPCs markedly increased cell numbers and were safe in teratoma formation assays [11].

Our previous studies and many others have demonstrated that MPCs can restore function in models of premature ovarian insufficiency [12–14] and various other diseases [11, 15, 16]. However, in a middle-aged female model undergoing natural aging, it was not determined whether hESC-MPCs benefit the long-term maintenance of reproductive fecundity and the ovarian reservoir or how their transplantation regulates ovarian function.

A recent study focused on preventing pathological changes in ovarian cells by mTOR inhibition, which postponed follicular activation and development and extended the follicle reserve and endocrine function of the ovaries [1]. Additionally, ovarian fibrosis in stromal interstitial tissues surrounding follicles is a key causal factor of obesity-induced ovarian dysfunction and the process is similar to that of reproductive aging [17]. Indeed, a reduction in adverse factors, inducing high levels of inflammation, reactive oxygen species (ROS), DNA damage, and apoptosis, could improve follicle quality and ovarian lifespan [18–21].

Furthermore, several studies have focused on the biomolecular interactions occuring in human bone marrow during aging. In addition to the remodeling of the mesenchymal stromal cell population in the bone marrow [22], changes in macrophages and macrophage-like immature cells (myeloid-derived suppressor cells, MDSCs) also occur during aging in healthy or tumor-bearing hosts [23]. MDSC comprise two large groups: granulocytic MDSCs (G-MDSCs), which are phenotypically and morphologically similar to neutrophils, and monocytic MDSCs (M-MDSCs), which are similar to monocytes [24]. These cells are precursors of innate immune cells, and their numbers increase with age [25]. It promotes age-related fibrosis and is involved in several immunosenescent processes. MDSCs have been found in many other abnormal conditions, such as autoimmunity, infection, diabetes and cardiac aging [26–30]. Several studies have reported that increased MDSC numbers contribute to the pathogenesis of these immune-aging-mediated diseases, and the transplantation of mesenchymal progenitor cells (MPCs) obtained from various sources has been shown to have beneficial effects in blocking or delaying disease progression through the suppression of inflammation and immune attack [31–33]. Immunosuppression derived from these two cell types (MSCs/MPCs and MDSCs) seems to have an opposite function in fibrosis, but the mechanism has not been well studied. It can be assumed that the secreted cytokines may cause this difference. Therefore, it is necessary to conduct further studies to reveal the detailed mechanisms.

In the present study, we found that multiple introductions of hESC-MPCs may contribute to preserving ovarian function in perimenopausal female mice by suppressing apoptosis and fibrosis and may maintain oocyte competence and delay reproductive senescence.

## Materials and methods

### Maintenance of hESC-MPCs

Human ESC-MPCs were differentiated and characterized as previously described [12, 14, 34]. Human CHA-hES15 ESCs (Korea Stem Cell Registry No. hES12010028) were mechanically detached using a glass pipette (Corning, Corning, NY) and cultured in a petri dish (Corning) for embryoid body (EB) formation. Fourteen days after EB formation, the cells were attached to culture dishes, and outgrowth cells were maintained in Dulbecco’s modified Eagle’s medium (DMEM)/low glucose (HyClone, Logan, UT) supplemented with 10% fetal bovine serum (FBS, Gibco, Franklin Lakes, NJ), 0.1 mM beta-mercaptoethanol (Invitrogen, Carlsbad, CA), 1% nonessential amino acids (NEAAs, Gibco), and 1% penicillin-streptomycin (P/S, Gibco). Sixteen days after EB attachment, the outgrowth cells were sub-cultured and maintained in DMEM/F12 medium (Gibco) supplemented with 10% FBS, 1% NEAA, 1% P/S, and 0.1 mM mercaptoethanol. These cells were defined as human ESC-MPCs and used in this study. The characteristics of human ESC-MPCs have been described previously [14, 34].

### Animal experiments

The animals were housed in the Animal Care Facility of CHA University according to the institutional guidelines for laboratory animals under temperature- and light- controlled conditions with a 12-h daily cycle and were fed *ad libitum*. The experimental protocols for animal use were approved by the Institutional Animal Care and Use Committee of CHA University (IACUC 190,050, 190,176, and 200,130). All experiments were designed and reported in accordance with the Animal Research: Reporting of In Vivo Experiments (ARRIVE) guidelines.

To prepare the natural perimenopausal mouse model, 6-month-old mice were purchased from KOATECH (Pyeongtaek, Gyeonggi-do, Korea) and maintained until 10 months of age. Next, 10-month-old female mice with a regular estrous cycles were randomly assigned to two groups: a phosphate-buffered saline (PBS)-injected control group (CON group) and an hESC-MPC-injected group (hESC-MPC group). The hESC-MPCs were intravenous injected every month for 4 months to delay ovarian aging. For the injection procedure of hESC-MPCs, mice were first anesthetized through the intraperitoneal injection of sterile avertin (tribromoethanol: 200 mg/10 ml/kg, Sigma-Aldrich, St. Louis, MO). To evaluate the reproductive performance of perimenopausal mice, 14-month-old female mice were caged individually with young males for mating. Pregnancy rates and mean litter sizes were recorded on Day 18.5 postconception. For sampling of tissues, blood, and fetus from experimental animals, mice were euthanized at the end of the study design point using CO_2_ infusion in a CO_2_ line-connected box at a gas infusion rate of 1.5–3.5 L/min. Once the animals cease respiration for 5–10 min, the euthanasia is confirmed by cervical dislocation.

### Estrous cycle analysis

Vaginal smears were collected in 100 µL of PBS at 9:00–10:00 every morning for 2 weeks. The smear samples were dropped onto a glass slides and air-dried on warm plates. The dried samples were stained with hematoxylin and eosin. The estrous cycle stages were determined as previously described [35]. Consistent cycles of proestrus (Pro), estrus (Est), metestrus (Met), and diestrus (Di) repeated every 4–5 days were considered a “regular estrous cycle” [35]. Irregular estrous cycles were defined as when the mice had at least one prolonged estrous cycle (> 5 days) before the end of the observation period. The experiments were repeated four times (*n* = 21 in the CON group; *n* = 27 in the hESC-MPC group), and the results are presented as the mean ± standard error of the mean (SEM).

### Ovarian follicle counting

Ovarian follicle counts ware performed as previously described [12]. Briefly, ovaries were fixed in 4% formaldehyde and embedded in paraffin. Each ovary was serially sectioned to 5 μm thickness and stained with hematoxylin and eosin to evaluate follicle growth. Follicle counts were performed on serially cut sections, counting every tenth section. Follicles (primordial, primary, secondary, and antral follicles and zona pellucida remnants) were classified and counted. Follicles were classified as previously described [36]. The percentage of follicles at each stage was calculated and compared between the groups. The results are expressed as the mean ± SEM. ( *n* = 6 in the CON group; *n* = 6 in the hESC-MPCs group).

### Enzyme-linked immunosorbent assay (ELISA)

Plasma was harvested, and the levels of 17β-estradiol (E_2_) and FSH were evaluated with ELISA kits (MyBioSource, San Diego, CA) according to the manufacturer’s instructions. Briefly, 50 µl of blank, standard or plasma sample was added to each well. Then 50 µl of biotin-labeled antibody working solution was added to each well. The microplate was incubated for 45 min at 37 °C. The plate was washed three times with wash buffer. Thereafter, 50 µl of HRP-streptavidin conjugate reagent was added to each well and incubated for 30 min at 37 °C. The plate was washed five times with the wash buffer. Then, 90 µl of TMB substrate was added to each well and incubated for 15 min at 37 °C in the dark. Then, 50 µl of stop solution was added to each well and the absorbance (O.D. 450) was measured and recorded by a microplate reader (Varian Company, Australia). (*n* = 21 in the CON group; *n* = 20 in the hESC-MPCs group).

### In vitro fertilization and embryo development

Epididymal sperm from 8 to 10-week-old males C57BL/6 N mice were collected in 500 µL of HTF medium (Millipore) and allowed to capacitate for 1 h before use. Cumulus-oocyte complexes (COCs) were inseminated for 4–5 h in a 50 µL drop of HTF medium and then the fertilized embryos were quickly washed in drops of KSOM medium (Millipore) by using a pasture pipette to remove unbound sperm and cumulus cells. Embryos were cultured in KSOM medium in a humidified atmosphere containing 5% CO_2_. The number of blastocysts was counted on day 5. Experiments were repeated at least five times.

### Bone marrow cell harvest and flow cytometry

Bone marrow (BM) was harvested from femurs by flushing them with RPMI medium. The cells were harvested by centrifugation at 800 g for 2 min and incubated in 1X RBC lysis buffer for 3 min. The cells were washed and harvested by centrifugation in PBS containing 1% FBS. The obtained BM cells were passed through a 70 μm cell strainer. The cells were incubated with fluorochrome-conjugated anti-mouse antibody for 1 h at 4 °C as follows. V450 mouse lineage antibody cocktail (BD biosciences), rat anti-mouse Ly-6 A/E (Sca-1)-PE-Cy™7 (BD biosciences), and rat anti-mouse CD117 (c-Kit)-APC (BD biosciences) antibodies were stained together to quantify the frequency of Lin^-^Sca1 + c-Kit^+^ (LSK) cells. A rat anti-mouse CD34-FITC antibody (BD biosciences) was used to further distinguish the LSK population from the long-term (LT) and short-term (ST) hematopoietic stem cell (HSC) populations. The MDSC population was identified with a combination of antibodies against CD11b-PE-Cy™7 (Invitrogen), Ly6G-FITC (Invitrogen) and Ly6C-APC (Invitrogen). Dead cells were excluded from the analysis by staining with 7-amino-actinomycin D (BD biosciences). The expression of the stained antibodies was measured using a FACSCanto II flow cytometer (BD Biosciences), and the acquired data were analyzed using FlowJo software (Tristar).

### Isolation of MDSCs and co-culture with hESC-MPCs in vitro

MDSCs were isolated from 6-month-old female mice splenocytes. Single cells were obtained by isolating splenocytes from the spleen and lysing red blood cells with Red Blood Cell Lysis Buffer (Biolegend), which was then filtered through a 70 μm cell strainer.

The isolated single splenocytes were separated into MDSCs using MACS. A Mouse MDSC Isolation Kit (Miltenyi Biotec) was used for MACS. The manufacturer’s instructions were isolated according to the MDSCs. For Ly6G + MDSCs separation, the cell pellet was resuspended in 350 µL MACS buffer (DPBS + 0.5% BSA + 2mM EDTA), FcR blocking reagent (50 µL) (Miltenyi Biotec) was added, mixed well, and incubated at 4 °C for 10 min. Then, 100 µL of biotin-conjugated anti-Ly6G antibody (Miltenyi Biotec) was added to the cells, which were then incubated for an additional 15 min at 4 °C. The cells were washed by centrifugation at 300 g for 10 min after adding 10 mL of MACS buffer. The labeled cells labeled were resuspended in 800 µL of MACS buffer, then 200 µL of anti-biotin microbeads (Miltenyi Biotec) were added and incubated for 10 min at 4 °C. The cells were washed with 20 mL MACS buffer and centrifuged at 300 g for 10 min. The cell pellet was resuspended in 500 µL of MACS buffer. Centrifugations was performed at 4 °C. The cells labeled with antibodies and microbeads underwent magnetic separation using LS columns (Miltenyi Biotec), following the instructions provided in the MDSC Isolation Kit (Miltenyi Biotec). The separated cells expressed the CD11b^+^Ly6G^+^ phenotype.

The isolated MDSCs were co-cultured with hESC-MPCs at a ratio of 5:1 (MDSCs:1 × 10^6^, hESC-MPCs:2 × 10^5^) in RPMI1640 medium (Gibco) for 72 h using a 24 well-transwell system. After co-culturing MDSCs in Transwell systems, the cells were counted and analyzed.

### Sirius red staining

Paraffin sections of ovarian tissue were exposed to xylene to remove the paraffin and rehydrated through sequential concentration changes starting with 100% ethanol, 70% ethanol, and finally, deionized water. Fibrotic areas in the ovarian tissue sections were detected using Sirius red staining (Abcam). The sections were incubated with picrosirius red solution targeting collagen for 60 min and then rinsed with acetic acid solution. The sections were dehydrated in absolute alcohol, and the slides were mounted and analyzed under a light microscope.

### Quantitative real-time PCR (qRT-PCR)

Total RNA isolation and cDNA synthesis were performed as described previously [37]. SYBR Green PCR master mix (Enzynomics, Daejeon, South Korea) was used, and all target genes were calculated using the comparative CT method. Expression levels of all genes were normalized to those of GAPDH. The primer sequences were *Gapdh*-F (TCAAGAAGGTGGTGAAGCAGG) *Gapdh-*R (CACATACCAGGAAATGAGCTT), *Il10*-F(TGGCCCAGAAATCAAGGAGC), *Il10*-R (CAGCAGA CTCAATACACACT), *Il6*-F (AGGATACCACTCCCAACAGACCT), *Il6*-R(CAAGTGCATCATCGTTGTTCATAC), *Tgf β1*-F (TGACGTCACTGGAGTTGTACGG) and *Tgf β1*-R (GGTTCATGTCATGGATGGTGC), *Arg1*-F (AGCACTGAG GAAAGCTGGTC) and *Arg1*-R (TACGTCTCGCAAGCCAATGT), *Col1a1*-F (CTGGCGGTTCAGGTCCAA T) and *Col1a1*-R (TTCCAGGCAATCCACGAGC), *Fibronectin*-F (CAACAACCGGAATTACACCG) and *Fibronectin*-R (GTCTCGGAGCTGGGAGTAGG).

### Statistical analysis

All experiments were repeated at least three times. The results are shown as the means ± SEM. The statistical significance of the differences was evaluated by one-way ANOVA with Duncan’s post-hoc test using SPSS ver. 18 software (SPSS Inc., Chicago, IL). Embryo development rates were analyzed by Student’s *t* test using GraphPad Prism 7.0 software (GraphPad Software, Inc., CA, USA). *P* values < 0.05 were regarded as statistically significant.

## Results

### hESC-MPCs contribute to maintaining estrus cycle regularity in perimenopausal mice

**Fig. 1:**
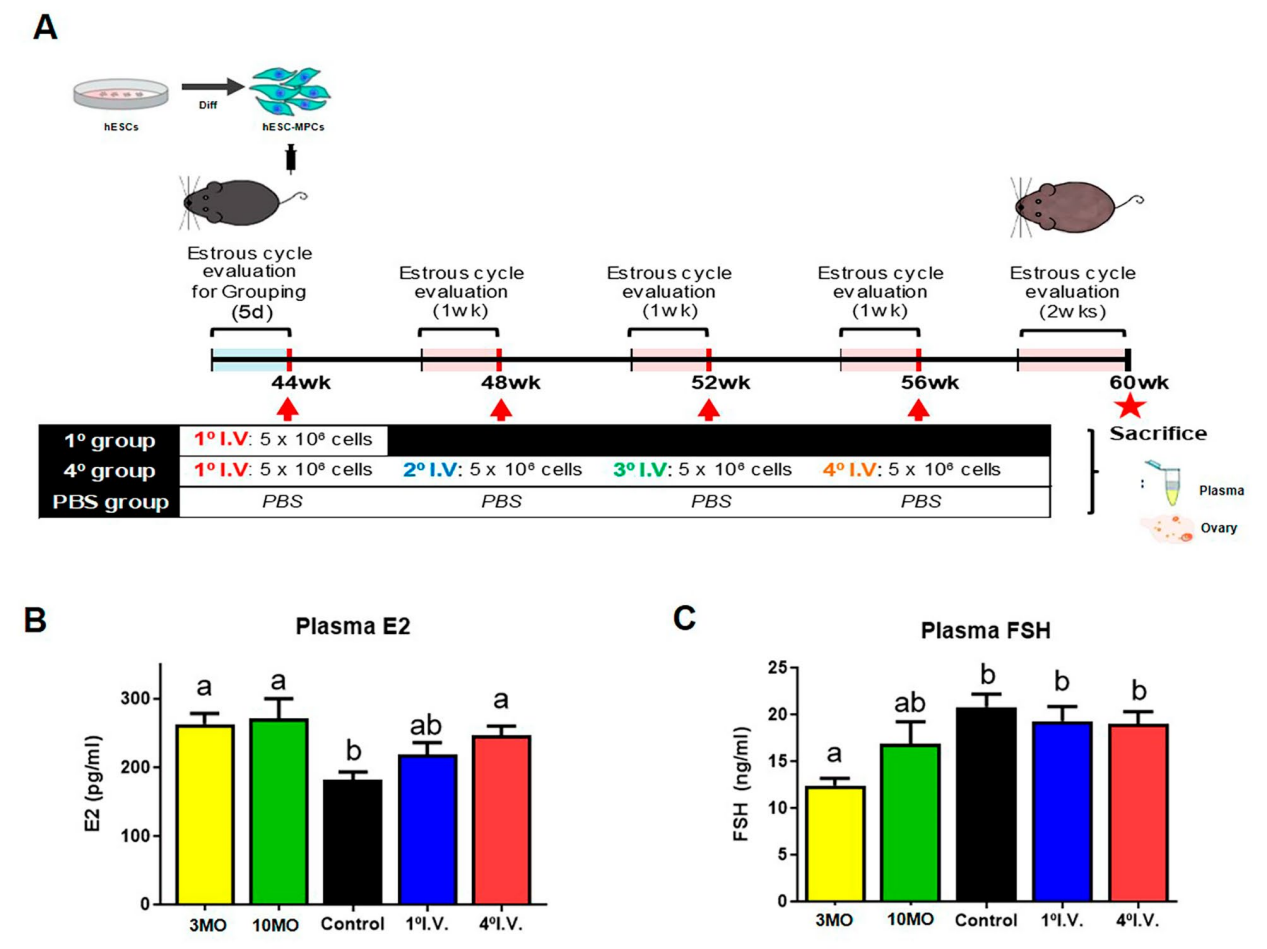
Effect of the number of human embryonic stem cell-derived mesenchymal progenitor cells (hESC-MPCs) on the plasma hormone levels in perimenopausal mice. **(A)** Experimental scheme of a preliminary experiment to determine the appropriate number of cell introductions. (**B** and **C**) The levels of sex hormones (serum 17β-estradiol (E2) and FSH) were measured using ELISA in plasma from various experimental groups (**3MO**, 3-month-old mice; **10MO**, 10-month-old mice; **control**, 14-month-old mice; **1° I.V.**, 14-month-old mice treated once with hESC-MPCs intravenously at 10 months of age; **4° I.V.**, 14-month-old mice treated monthly for 4 months with hESC-MPCs. Different superscript letters indicate significant differences (*p* < 0.05)

**Fig. 2:**
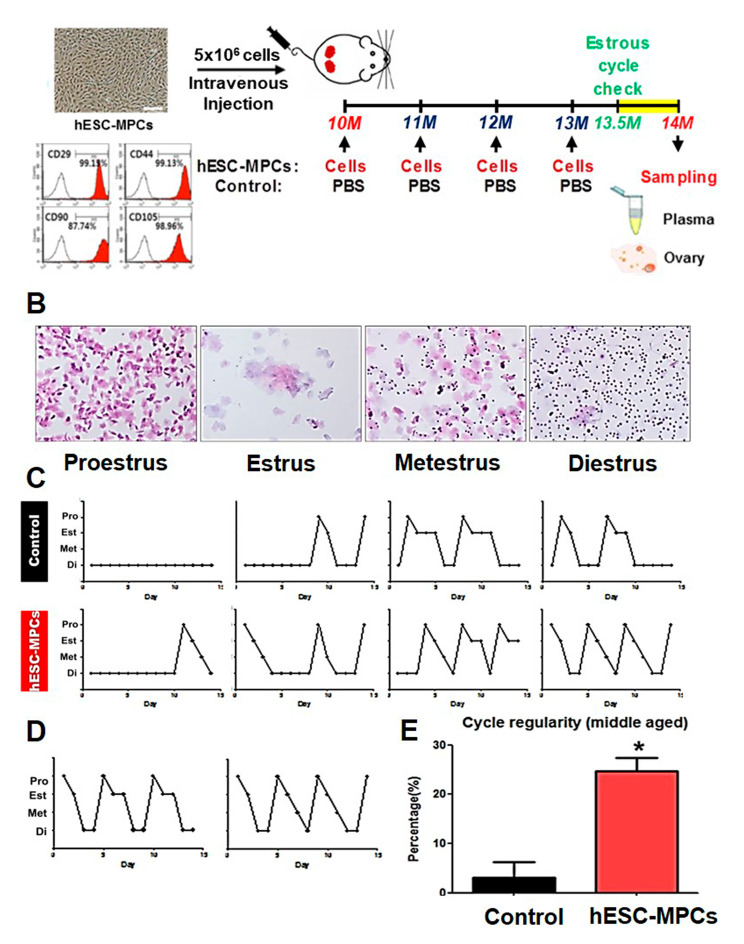
Effect of human embryonic stem cell-derived mesenchymal progenitor cells (hESC-MPCs) on the estrous cycles in perimenopausal mice. **(A)** Schematic illustrations of the experimental procedures used to evaluate the therapeutic effects of hESC-MPCs on reproductive aging in perimenopausal mice. **(B)** Representative images from hematoxylin-eosin (HE)-stained samples from vaginal lavage at each phase of the estrous cycle in C57BL/6 mice. **(C)** Staged estrus cycles of individual middle-aged control (14-month-old mice, upper) and hESC-MPC-multiple introduced mice (lower) (Pro, proestrus; Est, estrus; Met, metestrus; Di, diestrus). **(D)** Examples of regular estrous cycles within 2 weeks. **(E)** Estrus cycles were more regular in the hESC-MPCs group than in the control group as revealed by continuous vaginal smear continuous detection. *, significantly different (*p* < 0.05) (total *n* = 21 in the CON group; total *n* = 27 in the hESC-MPCs group). (total *n* = 20 in the CON group; total *n* = 21 in the hESC-MPCs group)

Reproductive cycles in middle-aged perimenopausal mice were analyzed after multiple-introductions of hESC-MPCs to evaluate the potential therapeutic function of these cells in delaying reproductive senescence. The cell introduction experiment was initiated in naturally aged mice at 10 months (44-weeks), which have ovarian function similar to that of human perimenopause [38]. Reproductive senescence was evaluated in mice at 14 months (60-weeks), which have an ovarian function similar to that during human menopause (Figs. 1A and 2A). Prior to the main experiments, we performed a preliminary experiment to determine the appropriate number of cells to introduce into a naturally aged mouse model (Fig. 1). The mice were maintained until 10 months of age (44 weeks of age), after which hESC-MPCs were introduced intravenously 1 time (1° I.V) and four times at monthly intervals (4° I.V). As shown in Fig. 1, the levels of sex hormones in the plasma showed a consistent pattern (plasma E2: 262.9 ± 15.6 pg/ml in 3-month-old, 271.1 ± 31.5 pg/ml in 10-month-old adult, 181.8 ± 8.2 pg/ml in 14-month-old control, 218.3 ± 8.3 pg/ml in 14-month-old 1° I.V, and 246.1 ± 12.5 pg/ml in 14-month-old 4° I.V; plasma FSH: 12.4 ± 0.8 ng/ml in 3-month-old, 16.9 ± 2.4 ng/ml in 10-month-old adult, 20.8 ± 1.4 ng/ml in 14-month-old control, 19.3 ± 1.5 ng/ml in 14-month-old 1° I.V, and 19.0 ± 1.3 ng/ml in 14-month-old 4° I.V). Therefore, we expected that multiple treatments of hESC-MPCs would have a better effect on fertility preservation in perimenopausal mice, and the 4° I.V group was chosen as the hESC-MPCs group (the mice were maintained until 10 months of age, and then hESC-MPCs were introduced intravenously at monthly intervals for 4 months) in the main study. Mice injected intravenously with saline (mock control) for the same duration were used as controls (Fig. 2A). The estrous cycles, consisting of 4 stages, proestrus (Pro), estrus (Est), metestrus (Met), and diestrus (Di), were checked (Fig. 2B and C) and consistent cycles of Pro, Est, Met, and Di repeated every 4–5 days were referred to as a “regular estrous cycle” (Fig. 2D) [39].

As reproductive aging can increase the length and irregularity of the estrous cycle, we evaluated the estrous cycle in 14-month-old mice with or without hESC-MPCs. Only 10-month-old mice with regular estrous cycles were included in the main study. As cycle regularity was extremely low in 14-month-old mice in the control group, age-matched mice in the hESC-MPCs group showed relatively high estrous cycle regularity (hESC-MPCs: 24.7 ± 2.7% vs. CON: 3.1 ± 3.1%, *p* < 0.05) (Fig. 2E), suggesting extension of fertility fecundity by hESC-MPCs.

### hESC-MPCs maintained ovarian structure and function in perimenopausal mice

**Fig. 3:**
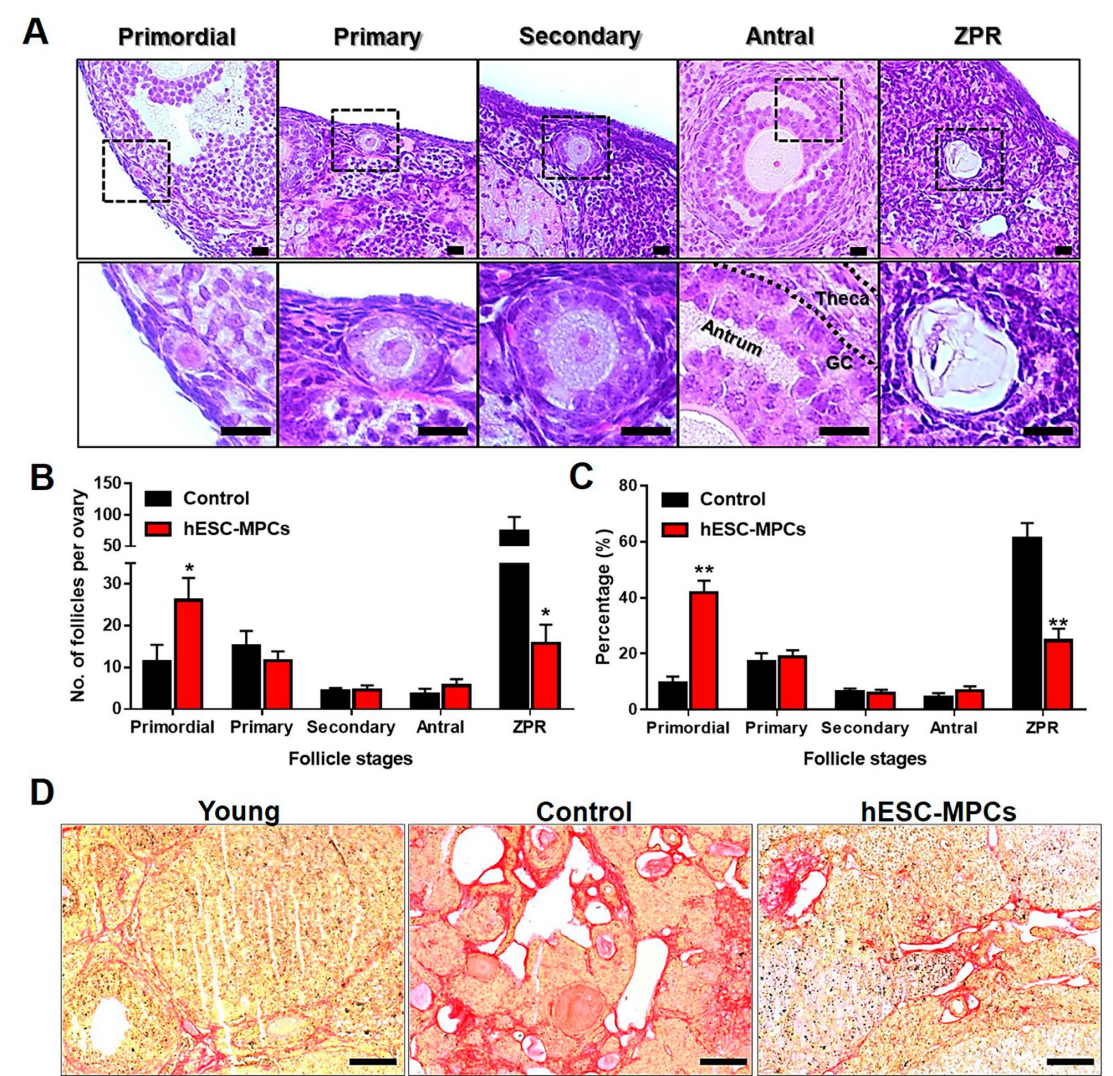
Maintenance of ovarian reserve by multiple introduction of human embryonic stem cell-derived mesenchymal progenitor cells (hESC-MPCs) in perimenopausal mice. **(A)** Follicles at different developmental stages in the ovaries. Scale bars = 20 μm. **(B)** Numbers of different types of follicles per ovary. **(C)** Quantification of the percentage of each follicle type per ovary. Significant differences are indicated (*p < 0.05, **p < 0.005). Theca, theca cell; GC, granulosa cell; Primordial, primordial follicle; Primary, primary follicle; Secondary, secondary follicle; Antral, antral follicle; ZPR, zona pellucida remnant. (total *n* = 6 in the CON group; total *n* = 6 in the hESC-MPCs group). **(D)** Representative images of picrosirius Red (PSR)-stained ovarian tissues from 3-month-old (young), 14-month-old (control), and 14-month-old mice treated monthly for 4 months with hESC-MPCs (hESC-MPCs). The intense red staining correlates with fibrotic regions

To further investigate whether hESC-MPC administration could delay the exhaustion of the ovarian reserve, histological analysis of the ovaries was performed (Fig. 3A). The number of primordial follicles representing ovarian reserve was significantly higher in the hESC-MPCs group (31.3 ± 8.2) than in the age-matched control group (19.2 ± 5.0). On the other hand, the number of zona pellucida remnants (ZPRs), which are considered atretic follicles, was much lower in the hESC-MPCs group (hESC-MPCs: 19.5 ± 6.8%, CON: 111.4 ± 33.3%, *p* < 0.05; Fig. 3B). To rule out the possibility that the difference in content occurred because of individual variations in ovarian volume, the ratio of each follicle count was normalized to the total number of follicles and compared (Fig. 3C). Interestingly, most follicles in the 14-month-old control group were ZPRs (51.7 ± 12.9); in contrast, the hESC-MPCs group had a significantly lower proportion of ZPRs (24.0 ± 4.6, *p* < 0.01). In addition, consistent with Fig. 3B, hESC-MPCs had a significantly higher proportion of primordial follicles than the 14-month-old control group (hESC-MPCs: 40.7 ± 5.6%, CON: 10.7 ± 3.2%, *p* < 0.01; Fig. 3C). These results suggest that hESC-MPC treatment can delay the exhaustion of ovarian reserve in even perimenopausal mice.

Several recent studies have reported that stromal fibrosis indicates ovarian aging [17, 40, 41]. We investigated whether hESC-MPC treatment reduced reproductive age-associated fibrosis in the ovaries. Picrosirius red (PSR), which stains connective tissue, especially collagen I and III fibers, was applied to the sampled ovaries. The fibrotic collagen fibers in the ovaries stained intensely red with PSR and increased in the 14-month-old control group (Fig. 3D). However, multiple treatments with hESC-MPCs reduced the age-dependent ovarian fibrosis. These results suggest that hESC-MPCs contribute to maintaining proper ovarian environment by reducing ovarian fibrosis, which may be associated with aging.

### hESC-MPCs improve the ovulation of mature eggs and embryo development in perimenopausal mice

**Fig. 4:**
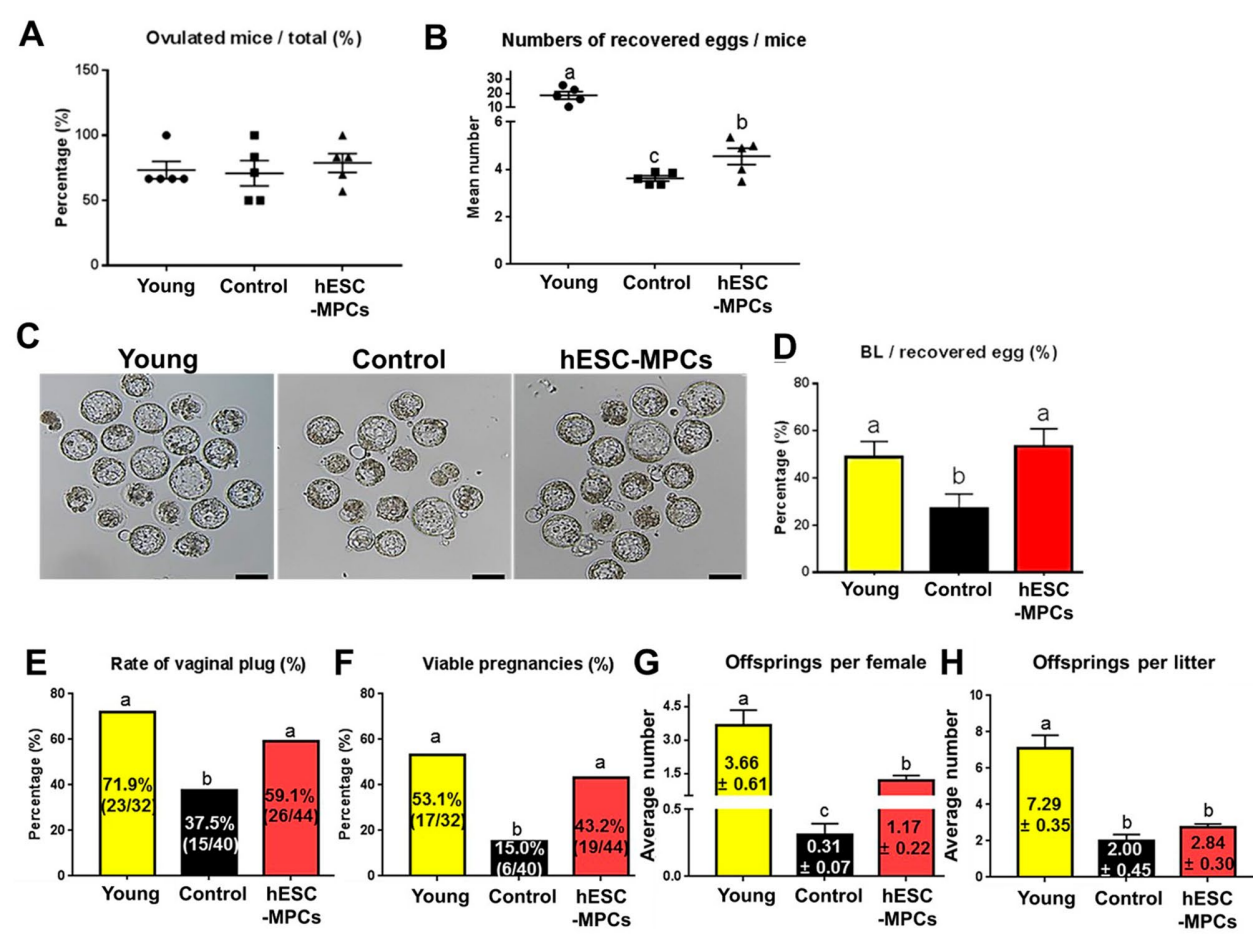
Effect of human embryonic stem cell-derived mesenchymal progenitor cells (hESC-MPCs) on ovulation, embryonic development, pregnancy and litter sizes in perimenopausal mice. **(A)** Ovulation rate, calculated as the number of ovulated mice per total mice. **(B)** The average number of eggs recovered per mouse. **(C)** Representative images of embryos after 120 h of culture *in vitro.* Scale bars = 50 μm. **(D)** Percentage of blastocyst formation after in vitro fertilization. **(E-H)** Female mice from the young group and experimental group were individually caged with a young male for mating. **(E)** Percentage of plug-positive mice per total mice. **(F)** Percentage of mothers that delivered viable fetuses per mouse. **(G)** Average number of offspring per mouse. **(H)** Average number of offspring per litter. Three-month-old (young), 14-month-old (control), and 14-month-old mice treated monthly for 4 months with hESC-MPCs (hESC-MPCs). Different superscript letters indicate significant differences (*p* < 0.05)

We investigated whether hESC-MPCs could improve ovulation in mature eggs and embryonic development, both of which are decreased by aging. Although the percentage of ovulated mice did not differ among the three groups (Fig. 4A), the number of recovered (ovulated) eggs per mouse was significantly reduced in both groups of 14-month-old mice (14-month-old control, 3.6 ± 0.1 and hESC-MPCs, 4.6 ± 0.3 group) compared to 3-month-old mice (18.7 ± 2.6; Fig. 4B). Furthermore, blastocyst formation rate was reduced with age (26.8 ± 6.4). In contrast, the hESC-MPCs group had significantly improved blastocyst formation (53.4 ± 7.4) compared with the 14-month-old control, to levels similar to that in the young control (48.7 ± 6.8; Fig. 4C and D).

### hESC-MPCs rescue poor reproductive outcomes in perimenopausal mice

To further investigate the therapeutic function of hESC-MPCs on pregnancy outcomes in perimenopausal mice, we evaluated reproductive parameters, including mating rate, delivery rate, number of offspring and litter size, in both groups after hESC-MPC therapy. In both groups, female mice were mated with young adult male mice. The values for all parameters were significantly lower in perimenopausal (14-month-old) mice than in young control (3-month-old) mice; (all values shown in Fig. 4). In contrast, hESC-MPC-treated mice had mating rates (Fig. 4E) and delivery rates (Fig. 4F) similar to the young controls. Although litter size was not significantly different between the 14-month-old control and hESC-MPC-treated mice (Fig. 4H), the hESC-MPC group had a significantly higher average number of offspring per female (Fig. 4G). These results suggest that fertility can be partially maintained by hESC-MPCs even with age.

### hESC-MPCs inhibit the increase in aging-related G-MDSCs and cellular inflammation in perimenopausal mice

**Fig. 5:**
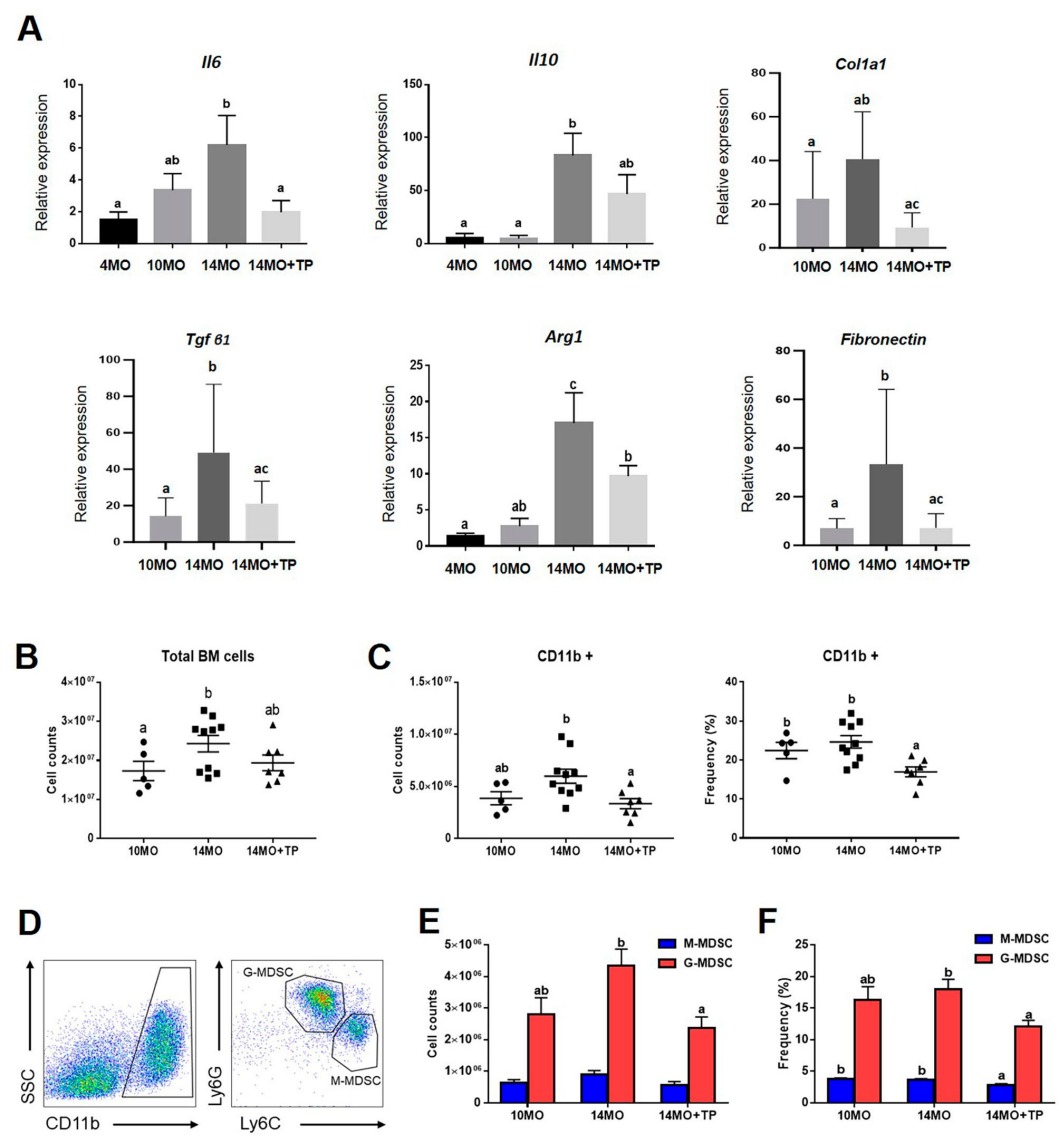
Effects of hESC-MPCs on the number of BM-derived MDSCs and inflammation and fibrosis-related genes in aged ovaries. **(A)** qRT-PCR analysis for of expression of pro-inflammatory cytokines (*Il6* and *Tgfβ1*), pro-fibrotic macrophage (*Il10* and *Arg1*) and pro-fibrotic genes (*Col1a1* and *Fibronectin*) in mouse ovaries from the 4-, 10- and 14-month-old groups treated/not treated with ESC-MPCs. **(B)** Total counts of BM cells from each group. **(C)** Cell counts and frequency of CD11b^+^ cells in BM. **(D)** Representative FACS plots of the G-MDSC (CD11b^+^Ly6G^hi^Ly6C^low^) and M-MDSC (CD11b^+^Ly6G^low^Ly6C^hi^) populations in BM. **(E-F)** Cell counts and frequency of G-MDSCs and M-MDSCs. Different superscript letters indicate significant differences (*p* < 0.05)

Aging-related chronic inflammation contributes to fibrosis and reduces organ function [42]. Thus, we examined the expression levels of genes related to inflammation and fibrosis in the ovaries to determine whether ovarian fibrosis, which is associated with aging, is related to an inflammation-mediated response [17, 43–45]. As shown in Fig. 5A, the expression of pro-inflammatory cytokines (*Il6* and *Tgfβ1*) was significantly upregulated in the 14-month-old mouse group compared to young and 10-month-old mice groups. Furthermore, the expression of profibrotic macrophage genes (*Il10* and *Arg1*) and representative fibrosis markers, such as *Col1a1* and *Fibronectin*, were significantly upregulated. Importantly, the introduction of hESC-MPCs effectively reduced the expression of these pro-inflammatory cytokines and fibrosis mediators. The total number of BM cells was significantly higher in 14-month-old mice than in 10-month-old mice (Fig. 5B). In addition, the number and frequency of CD11b + cells, which may be monocytes, macrophages or granulocytes, were significantly decreased by transplantation of hESC-MPCs (Fig. 5C). These findings suggested that pro-inflammatory and pro-fibrotic changes in aged mouse ovaries may be related to monocyte-derived immune cells in the BM.

As individuals age, the number of MDSCs increases in humans and mice [29]. Immunosenescence also affects MDSCs in aged mice, and MDSCs in aged mice promotes inflammation and fibrosis [25, 26, 30]. Therefore, we investigated whether transplantation of hESC-MPCs into aged mice affected the number and frequency of MDSCs in the BM. Mouse MDSCs were classified into two types based on the degree of Ly6G and Ly6C expression of CD11b as follows: CD11b^+^Ly6G^hi^Ly6C^low^ (G-MDSCs) and CD11b^+^Ly6G^low^Ly6C^hi^ (M-MDSCs) (Fig. 5D). We found that the number and frequency of G-MDSC subsets in 14-month-old-mice were significantly reduced by the transplantation of hESC-MPCs (Fig. 5E and F). Furthermore, in vitro, we demonstrated the effect of hESC-MPCs on the suppression of MDSCs expansion during co-culture. G-MDSCs (CD11b^+^Ly6G^+^) were isolated from the spleens of 6-month-old mice by MACS and co-cultured with hESC-MPCs. The isolated G-MDSCs isolated had a purity greater than 96% (Supplementary Fig. 1A). When co-cultured with hESC-MPCs in a transwell system for 72 h, MDSCs expansion was significantly suppressed (Supplementary Fig. 1B). These results suggest that hESC-MPCs suppress the expansion of MDSCs with age and have beneficial anti-inflammatory and antifibrotic effects on aging ovaries.

## Discussion

In the present study, we found that multiple introductions of hESC-MPCs may delay ovarian senescence and maintain ovarian reserve to make good embryos in perimenopausal female mice by reducing inflammation and fibrosis, and that restoring ovarian environment by stem cell-based cell therapy can contribute to preserving ovarian function.

Recently, stem cell therapy using mesenchymal progenitor cells/mesenchymal stem cells (MPCs/MSCs) from various adult or fetal tissues has been used to achieve a therapeutic effects and inhibit reproductive senescence [6, 46, 47]. hESC-MPCs have been suggested as an alternative to tissue-derived MPCs due to their high proliferative capacity and ease of standardization, but their therapeutic properties for maintaining ovarian function in perimenopausal females have not been determined.

Menopause is defined as the cessation of the menstrual cycle for 12 months or more and is mainly caused by decreased secretion of 17β-estradiol [48]. Menopause can be induced by surgery or cancer treatments [49]. As women age, their menstrual cycle becomes less regular, and the amount of FSH increases in response to reduced levels of ovarian hormones. This perimenopause usually begins in women between 35 and 45 years of age and menopause occurs between 47 and 51 years of age [50]. Because human perimenopause occurs around the age of 38 years, according to previous calculation methods [18, 38], it can be expected that reproductive senescence will occur in mice from approximately 10 months of age. The mean age of menopause is approximately 51 years in women and 15 months in mice. As shown in Fig. 2C-E, the rate of regular estrous cycles was significantly higher in the hESC-MPC-injected group compared with the control group, and the levels of E_2_ and FSH in the hESC-MPC-injected group were more similar to those in the young adult group than the middle-aged control group (Fig. 1B and C). In addition, the percentage of primordial follicles was significantly higher in the treated group than that in the age-matched control group. A significantly decreased number and proportion of ZPRs (degenerating follicles) were found in the hESC-MPC group compared to the control group (Fig. 3B and C). Consistent with these observations, not only reproductive performance but also the quality of blastocysts of perimenopausal mice were improved by hESC-MPC injections (Fig. 4). Based on these results, we have confirmed the therapeutic potential of hESC-MPCs for preserving ovarian reservoir and extending fertility fecundity in perimenopausal mice.

Recent studies have reported that various adverse factors that can induce inflammation, ROS, DNA damage, and apoptosis decrease follicle quality and ovarian lifespan [19–21]. For example, the levels of the proinflammatory cytokine IL-1a are enriched in developing follicles, and their ovarian mRNA levels increase with age. In fact, IL-1a knockout significantly increased female fertility in 12-month-old mice [21]. Additionally, increased collagen deposition, indicative of tissue fibrosis, has been documented in the ovaries of postmenopausal women and animal models of reproductive aging [40, 51]. Targeted removal of fibrotic collagen from mouse ovaries may extend female reproductive lifespan [17]. Therefore, the cellular events that cause ovarian fibrosis should be determined. In the present study, we observed an age-dependent increase in ovarian fibrosis, which was reduced after multiple introductions of hESC-MPCs (Fig. 3D). To identify the regulators of hESC-MPC-mediated ovarian fibrosis, we analyzed the status of bone marrow components among the experimental groups. The cell counts of the BM and HSCs were similar in all groups. However, the number of MDSCs in the hESC-MPC group was lower than that in the control group (Fig. 5B and F). Several studies have verified the expansion of MDSCs with age [23, 52] and that MDSCs play a role in inducing aging-related cardiac fibrosis [26]. In addition to increased numbers of MDSCs, there were a significant change in the levels of pro-inflammatory (*Il6 and Tgf β1*) and pro-fibrosis (IL10 and Arg1) cytokine molecules in the ovaries in the hESC-MPC group (Fig. 5A). In previous studies, intravenously delivered hESC-MPCs had restored cisplatin-induced ovarian damage by reducing apoptotic signaling in stromal cells and increasing granulosa cell proliferation [14]. Additionally, by local delivery of these cells into the same mouse model, we found that the secretome of hESC-MPCs may help prevent ovarian degeneration and retain female fecundity [12]. Therefore, in the present study, we identified the anti-inflammatory and anti-fibrotic roles of ES-MPC-based cell therapy in the ovarian environment through the suppression of MDSCs. Also, it would be suggested that long-term exposure of hESC-MPCs may have positive effects on maintaining ovarian function in perimenopausal mice undergoing natural aging. However, further studies are needed to reveal the mechanisms by which ESC-MPCs regulate MDSC proliferation and its related factors.

## Conclusions

From these results, we suggests that multiple introductions of hESC-MPCs may improve ovarian environment and reservoir by reducing inflammation and fibrosis via downregulation of MDSC production in BM, and it extend fertility and fecundity in perimenopausal female mice. Therefore, hESC-MPCs could be a useful sources for the cell therapy to preserve ovarian function in naturally aging females. Also, the development of a safe introduction method for patients is required for clinical application.

### Electronic supplementary material

Below is the link to the electronic supplementary material.

Supplementary Material 1: Fig. 1. Impact of hESC-MPCs on MDSCs expansion in an in vitro system. (A) Representative FACS plot of MDSCs isolated from the spleen. (B) MDSC cell numbers after co-culture with hESC-MPCs. (*p < 0.05).

## Data Availability

The authors declare that the dataset supporting the conclusions of this study has been included in the article. Data supporting the findings of this study are available from the corresponding author upon request.

## Declarations

The experimental protocols were approved by the Institutional Animal Care and Use Committee of CHA University (title of the approval project: A study for improvement of ovarian reproductive function using human embryonic stem cell-derived mesenchymal progenitor cells; name of the institutional approval committee: CHA University IACUC; Approval number and date: IACUC 190050 (July 24, 2018), IACUC 190176 (July 31, 2019), and IACUC 200130 (Aug. 18, 2020)).

## Abbreviations

ESC: Embryonic stem cell
MPC: Mesenchymal progenitor cell
G-MDSC: Granulocytic myeloid-derived suppressor cell
M-MDSC: Monocytic myeloid-derived suppressor cell
mTOR: Mammalian target of rapamycin
ROS: Reactive oxygen species
EB: Embryoid body
DMEM: Dulbecco’s modified Eagle’s medium
FBS: Fetal bovine serum
IACUC: Institutional Animal Care and Use Committee
ELISA: Enzyme-Linked Immunosorbent Assay
HRP: Horseradish peroxidase
TMB substrate: 3,3′,5,5′-Tetramethylbenzidinesubstrate
HTF medium: Human tubal fluid medium
COCs: Cumulus-oocyte complexes
KSOM medium: Potassium-supplemented simplex optimised medium
BM: Bone marrow
LSK: Lin^−^Sca1 + c-Kit^+^
I.V.: Intravenous
WT: Wild type
E2: Estradiol
FSH: Follicle stimulating hormone
ZPR: Zona pellucida remnants
PSR: Picrosirius red
