# Supplementary figures and images for: Multiple treatments with human embryonic stem cell-derived mesenchymal progenitor cells preserved the fertility and ovarian function of perimenopausal mice undergoing natural aging
Source: Stem Cell Res Ther. 2024 Mar 3;15:58. doi: 10.1186/s13287-024-03684-6 (PMC10910829; doi:10.1186/s13287-024-03684-6)

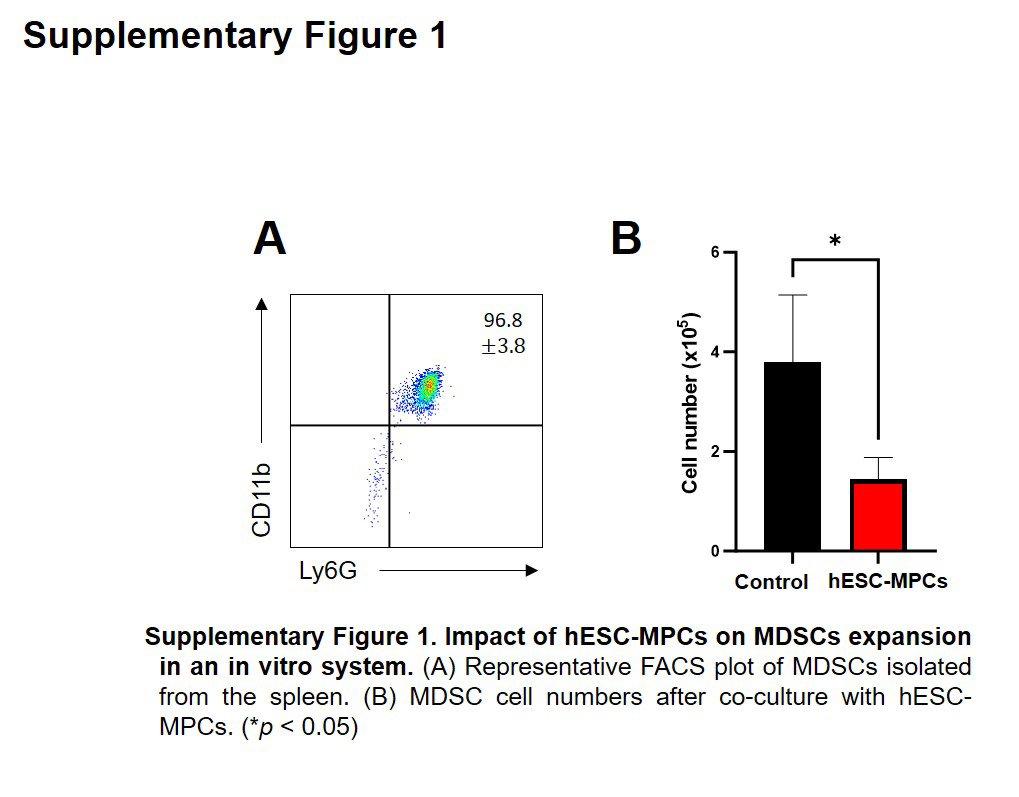

Supplement: Supplementary file 1 — Supplementary Material 1: Fig. 1. Impact of hESC-MPCs on MDSCs expansion in an in vitro system. (A) Representative FACS plot of MDSCs isolated from the spleen. (B) MDSC cell numbers after co-culture with hESC-MPCs. (*p < 0.05). [file 13287_2024_3684_MOESM1_ESM.tif]
